# Supplementary material for: Transcriptome and Metabolome Analyses Provide Insight into the Glucose-Induced Adipogenesis in Porcine Adipocytes
Source: Curr Issues Mol Biol. 2024 Mar 3;46(3):2027–42. doi: 10.3390/cimb46030131 (PMC10968871; doi:10.3390/cimb46030131)
Supplement: Supplementary file 1 [file cimb-46-00131-s001.zip › FigureS1-S3.pdf]

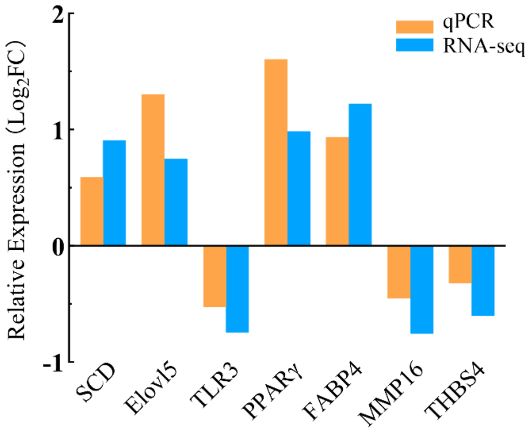

Figure S1. Validation of seven randomly selected DEGs by RT-qPCR.

A

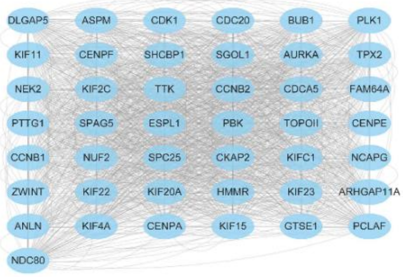

Category	Gene name in MCODE1	N	P-value
**GO Biological Process**			
Mitotic cell cycle	GTSE1, ESPL1, KIFC1, NDC80, KIF2C, CDC20, TTK, CCNB2, KIF23, NUF2, TPX2, CENPA, NCAPG, PBK, CDK1, KIF11, SGOL1, KIF4A, CDCA5, KIF20A, CENPF, NEK2, SPC25, ANLN, TOPOII, SPAG5, ZWINT, CCNB1, PLK1, KIF22, CENPE, BUB1, AURKA	33	3.22×10^{-39}
**KEGG Pathway**			
Oocyte meiosis	ESPL1, CDC20, CCNB2, CDK1, SGOL1, PTTG1, CCNB1, AURKA	8	6.59×10^{-9}
Cell cycle	ESPL1, CDC20, CCNB2, CDK1, PTTG1, CCNB1	6	8.81×10^{-6}

B

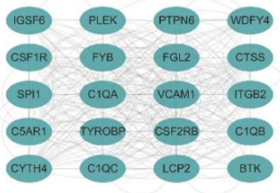

Category	Gene name in MCODE2	N	P-value
**GO Biological Process**			
Immune system process	TYROBP, C1QA, C1QC, C1QB, CTSS, VCAM1, PLEK, CSF1R, FGL2, FYB, LCP2, PTPN6, BTK, IGSF6, C5AR1	15	8.498×10^{-9}
**KEGG Pathway**			
Complement and coagulation cascades	C1QA, C1QC, C1QB, C5AR1, ITGB2	5	1.64×10^{-6}

Figure S2. Function and pathway enrichment analyses of Module 1 (A) and Module 2 (B).

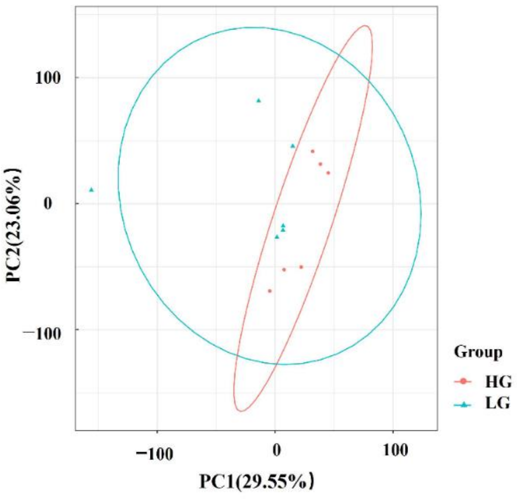

Figure S3. Principal component analysis (PCA) of the metabolite profiles for LG and HG groups
