# Supplementary material for: Transcriptome and Metabolome Analyses Provide Insight into the Glucose-Induced Adipogenesis in Porcine Adipocytes
Source: Curr Issues Mol Biol. 2024 Mar 3;46(3):2027–42. doi: 10.3390/cimb46030131 (PMC10968871; doi:10.3390/cimb46030131)
Supplement: Supplementary file 1 [file cimb-46-00131-s001.zip › Table S1. Primers used for experimtal validation by qRT-PCR analysis.pdf]

Table S1 Primers used for experimtal validation by qRT-PCR analysis

Gene name[1]	Accession number	Primer		Size(bp)
		Direction	Sequence (5' to 3')	
MMP16	XM_001926617.6	F	CAGAAGTGGCAGCACAAAC	241
		R	ATCCTCCCTCTCCATCAAA	
SCD	XM_021072070	F	TGCTGATCCCCACAATTCCC	352
		R	CTCCCGGGGGCTAATAGTCT	
Elovl5	XM_021098832	F	TGTGAGTTGTGCCCAATGCT	166
		R	CTGATGGAAGAGGTCCTACGG	
FABP4	NM_001002817	F	GACAGGAAAGTCAAGAGCACC	228
		R	CGGGACAATACATCCAACAGAGT	
TLR3	NM_001097444.1	F	ACAAAACCAGCAACACGAC	187
		R	TTGGAAAGCCCATAAAGAG	
PPARγ	NM_214379.1	F	TTATTGACCCAGAAAGCG	88
		R	AGGATTTCATACCGCAGG	
THBS4	XM_021084490.1	F	TTGTGACGAGGATGCTGAT	259
		R	GTCTTGCTGGTTACGATTG	
β-actin	XM_003124280	F	GATCGTGCGGGACATCAA	180
		R	AGGAAGGAGGGCTGGAAGAG	

[1]MMP16, matrix metallopeptidase 16; SCD, stearoyl-CoA desaturase; Elovl5, fatty acid elongase-5; FABP4, fatty acid-binding protein 4; TLR3, toll like receptor 3; PPARγ, peroxisome proliferator-activated receptor gamma; THBS4, thrombospondin-4.
