# Supplementary material for: Transcriptome and Metabolome Analyses Provide Insight into the Glucose-Induced Adipogenesis in Porcine Adipocytes
Source: Curr Issues Mol Biol. 2024 Mar 3;46(3):2027–42. doi: 10.3390/cimb46030131 (PMC10968871; doi:10.3390/cimb46030131)
Supplement: Supplementary file 1 [file cimb-46-00131-s001.zip › Table S2. Output statistics of sequencing data.pdf]

Table S2. Output statistics of sequencing data

Samples	Clean reads	Clean bases	GC content (%)	Q30 (%)	Mapped Reads	Uniq Mapped Reads
LG1	26,178,107	7,833,355,028	52.93%	95.01%	50,328,338 (96.13%)	48,858,519 (93.32%)
LG2	26,872,283	8,035,194,410	52.81%	94.96%	51,776,330 (96.34%)	50,360,805 (93.70%)
LG3	26,258,631	7,856,750,622	52.63%	94.91%	50,510,536 (96.18%)	49,063,069 (93.42%)
LG4	23,809,411	7,125,046,442	52.82%	95.02%	45,693,418 (95.96%)	44,463,527 (93.37%)
HG1	25,138,045	7,521,646,898	52.69%	95.01%	48,347,927 (96.16%)	47,074,542 (93.63%)
HG2	23,400,493	7,002,757,020	52.72%	94.92%	45,241,448 (96.67%)	44,094,965 (94.22%)
HG3	24,369,010	7,294,670,124	52.78%	95.26%	47,288,422 (97.03%)	46,127,705 (94.64%)
HG4	24,700,404	7,383,674,688	53.05%	94.96%	47,428,976 (96.01%)	45,992,578 (93.10%)
